# Retinal Degeneration and Visual Outcomes in Patients With Bardet-Biedl Syndrome: Genotypic Influences From a Caribbean Cohort

**DOI:** 10.7759/cureus.99380

**Published:** 2025-12-16

**Authors:** Sebastián J Vázquez-Folch, Gabriel A Jiménez-Berríos, Natalio Izquierdo, Omar Garcia Rodriguez

**Affiliations:** 1 School of Medicine, Universidad Central del Caribe, Bayamón, PRI; 2 Department of Ophthalmology, School of Medicine, Medical Sciences Campus, University of Puerto Rico, San Juan, PRI; 3 Department of Epidemiology, Moffitt Cancer Center, Tampa, USA

**Keywords:** bardet-biedl syndrome, ciliopathy, puerto rico, retinal degeneration, rod-cone retinal dystrophy

## Abstract

Background

The Bardet-Biedl syndrome (BBS) is a rare multisystem ciliopathy characterized by retinal degeneration, polydactyly, truncal obesity, hypogonadism, and hypogonadotropism. Progressive rod-cone dystrophy leads to early-onset vision loss. Despite increased genetic screening, BBS remains underdiagnosed in Caribbean populations. This study aims to describe the genetic and ocular phenotype of patients with BBS in Puerto Rico and explore genotype-phenotype correlations.

Methods

We conducted a retrospective chart review of 36 genetically confirmed BBS patients from a hereditary retinal disease clinic in Puerto Rico. Data collected included best-corrected visual acuity (BCVA), refractive error, visual field mean deviation (MD), and macular optical coherence tomography (OCT) measurements (volume and thickness). Genetic testing identified the BBS subtype and zygosity. Descriptive and statistical analyses were performed. The study was approved by a certified institutional review board (IRB).

Results

Age ranged from three to 69 years old. Mutations in the BBS1 gene were most frequent (86%), followed by BBS7 (8%), BBS10 (3%), and BBS19 (3%). Most patients had homozygous mutations in the BBS1 (%). The mean BCVA was 2.0 logMAR OD and 2.1 logMAR OS. Patients with homozygotic mutations in the BBS1 had worse vision than compound heterozygotes (p=0.025). Upon visual field examination, the MD was -27.6 dB OD and -28.3 dB OS. Near-absent visual fields occurred in patients with advanced disease. Macular volume and thickness averaged 8.2 mm³/227 µm (OD) and 7.7 mm³/216 µm (OS). Structural damage was associated with inner/outer segment disruption on OCT.

Conclusions

Mutations in the BBS1 are the most frequent in Puerto Rico, often associated with profound visual impairment and retinal thinning. These findings underscore the importance of early ophthalmic evaluation and genetic testing in patients with syndromic retinitis pigmentosa. Genotype-specific differences in visual decline support personalized surveillance and future therapeutic planning.

## Introduction

The Bardet-Biedl syndrome (BBS) is an autosomal recessive ciliopathy characterized by a spectrum of systemic and ocular manifestations, including rod-cone retinal dystrophy, polydactyly, truncal obesity, hypogonadism (often hypogonadotropic), and cognitive impairment [1,2]. Progressive retinal degeneration is the most consistent and often the earliest feature, occurring in up to 90% of affected individuals and leading to progressive vision loss and, ultimately, legal blindness [3].

To date, more than 20 genes have been implicated in BBS, with BBS1 being among the most frequently mutated [4]. In Puerto Rico, a genetically isolated population, evidence suggests an especially high prevalence of BBS1 mutations, with a distinctive allele distribution compared to other populations [5]. While several genotype-phenotype studies have suggested that BBS1 variants may be associated with milder systemic and ocular outcomes compared to BBS7 or BBS10 mutations [6], certain alleles (such as p.Glu549) have been linked to more severe clinical presentations [7]. Notably, homozygous BBS1 mutations have been correlated with significantly reduced visual acuity and pronounced macular thinning [8,9].

Despite advances in genetic testing, BBS remains underdiagnosed in the Caribbean, including Puerto Rico, and comprehensive genotype-phenotype correlation studies in this region remain limited. A deeper understanding of how specific BBS genotypes, particularly zygosity status, influence ocular structure and function is critical for improving prognostic accuracy, guiding genetic counseling, and informing future therapeutic development.

In this study, we characterize the ophthalmic and genetic profiles of 36 Puerto Rican patients with BBS using multimodal data, including visual acuity, visual fields, refractive error, and macular optical coherence tomography (OCT). We specifically investigate the relationship between genotype and the severity of retinal degeneration, with the goal of enhancing clinical management and supporting future interventional strategies.

## Materials and methods

This retrospective observational study was conducted at Hospital San Francisco in San Juan, Puerto Rico. The patients' medical records with a genetically confirmed diagnosis of BBS were evaluated between January 2010 and December 2023. Eligible patients were required to have a molecular diagnosis of BBS confirmed by either targeted gene panel testing or whole-exome sequencing (Invitae, San Francisco, California, United States), as well as completion of a comprehensive ophthalmic examination that included best-corrected visual acuity (BCVA), refraction, visual field testing (Humphrey 30-2), and OCT. Only patients with complete multimodal ophthalmic imaging, specifically macular OCT and reliable visual field testing, were included; the absence of fundus autofluorescence or other ancillary imaging modalities was not considered an exclusion criterion. Patients were excluded if their records lacked complete ophthalmic data or if they had additional ocular comorbidities that could confound the retinal measurements. Electrophysiologic testing, including electroretinogram (ERG), was not consistently available at our center during portions of the study period and was therefore not required for inclusion.

Genetic testing was done using next-generation sequencing (NGS) through Invitae. The testing panel included all currently known genes associated with BBS. Patient genotypes were classified by causative gene (e.g., BBS1, BBS7, BBS10, BBS19) and by zygosity status, which was further categorized as homozygous, heterozygous, or compound heterozygous.

Comprehensive ophthalmic examinations were done. BCVA was measured with a Snellen chart and subsequently converted to logarithm of the minimum angle of resolution (logMAR) values for statistical analysis. Cyclopegic refraction was recorded, and the spherical equivalent was calculated. Anterior segment slit lamp examination and indirect ophthalmoscopy were done. Macular OCT scans were analyzed for central macular thickness and macular volume using Carl Zeiss Meditec, Inc. Cirrus 6000 (Dublin, California, United States) with the standard macular cube 512×128 protocol.

Because BBS1 exhibits notable genotype-phenotype variability, particularly between homozygous and compound heterozygous variants, these two groups were selected for comparative analysis. Descriptive and inferential statistical analyses, including chi-squared and Kruskal-Wallis tests, were performed. Data distribution was assessed using the Shapiro-Wilk test to guide the selection of parametric versus non-parametric analyses. Given the exploratory nature of the study and the limited sample size, corrections for multiple comparisons were not applied. The analyses aimed to compare visual acuity, macular thickness, macular volume, and refraction between BBS1 compound heterozygous and BBS1 homozygous individuals. A p-value <0.05 was considered statistically significant. Any missing values were excluded case-by-case.

Verbal informed consent was obtained from all participants at the time of clinical evaluation. All data were de-identified prior to analysis to ensure patient confidentiality. The study protocol was approved by the Institutional Review Board of Universidad Central del Caribe (approval number: 2025-36).

## Results

Demographics and genetic findings 

A total of 36 patients with genetically confirmed BBS were included. Patient ages ranged from three to 69 years (mean±SD: 34.7±18.2 years). There was a slight male predominance (20 males, 55.6%) (Table 1).

**Table 1:** Sex and age distribution BBS: Bardet-Biedl syndrome

	BBS1	BBS7, BBS10, and BBS19
Male	16	4
Female	15	1
Mean age	32.3	21

Table 2 summarizes the distribution of causative genes by zygosity in the 36 Puerto Rican patients with BBS. The majority of patients carried BBS1 mutations (86.1%), most frequently in the homozygous state (58.3%). Compound heterozygosity was observed in 38.9% of the cohort, while only one patient (2.8%) was heterozygous. Less common genotypes included BBS7 (8.3%), BBS10 (2.8%), and BBS19 (2.8%).

**Table 2:** Gene distribution by zygosity BBS: Bardet-Biedl syndrome

Zygosity	BBS1 n (%)	BBS10 n (%)	BBS19 n (%)	BBS7 n (%)
Compound heterozygous	13 (92.9)	1 (7.1)	0 (0)	0 (0)
Heterozygous	0 (0)	0 (0)	0 (0)	1 (100)
Homozygous	18 (85.7)	0 (0)	1 (4.8)	2 (9.5)
Total	31 (86.1)	1 (2.8)	1 (2.8)	3 (8.3)

Patients with homozygous BBS1 mutations demonstrated worse visual acuity compared to compound heterozygotes. The mean BCVA was 2.27 logMAR in the homozygous group versus 1.91 logMAR in the compound heterozygous group. This difference was statistically significant in the right eye (mean difference 0.36 logMAR; 95% CI: 0.04-0.68; p=0.026; chi-squared value: 4.99), though it did not reach significance in the left eye (p=0.06; chi-squared value: 3.51). These findings highlight the effect of zygosity on visual severity within the BBS1 subgroup.

Visual acuity and refraction 

The mean BCVA was 2.0 logMAR in the right eye and 2.1 logMAR in the left eye, consistent with legal blindness. Patients with homozygous BBS1 mutations demonstrated significantly worse BCVA compared to compound heterozygotes (p=0.026; chi-squared value: 4.99), in the right eye. Refractive errors varied across the cohort, with a high prevalence of myopia and astigmatism. The mean spherical equivalent was -1.05±2.62 D in the right eye (OD) and -1.01±2.66 D in the left eye (OS), with a high prevalence of myopia and astigmatism. Overall, 21.6% of patients had high myopia (≤-6.00 D).

Refractive error

When comparing refractive errors in BBS1 patients with BBS7, BBS10, and BBS19 patients, no statistical significance was found. The mean spherical equivalent for patients with BBS1 was -0.90 D in the right eye and -0.83 D in the left eye, while patients with BBS7, BBS10, and BBS19 had a mean spherical equivalent of -1.85 D and -1.95 D in the right and left eyes, respectively. 

Macular volume

Patients with BBS1 had a mean macular volume of 8.00 mm³ OD and 7.74 mm³ OS, whereas patients with BBS7, BBS10, and BBS19 had a mean macular volume of 9.58 mm³ OD and 7.10 mm³ OS. There were no statistically significant differences in macular volume between patients with both types of the syndrome in either eye.

Macular thickness

The mean macular thickness in patients with BBS1 was 222.00 µm OD and 219.00 µm OS, compared to 265.50 µm OD and 197.25 µm OS in patients with BBS7, BBS10, and BBS19. There was no statistically significant difference in macular thickness between these groups of patients in either eye.

## Discussion

This study provides a detailed characterization of retinal degeneration and genotype-specific visual outcomes in a cohort of Puerto Rican patients with BBS. Our findings show a high prevalence of BBS1 mutations (86%), with the majority occurring in a homozygous state. Visual function was profoundly impaired, with the mean BCVA in the range of legal blindness and marked visual field constriction across nearly all patients.

These results are consistent with prior studies reporting BBS1 as the most frequently mutated gene in BBS, particularly in European and North American cohorts [3,10,11]. However, the frequency observed in our cohort was even higher, suggesting the presence of a founder effect in Puerto Rico. This aligns with prior observations by Guardiola et al., who reported an unusually high prevalence of BBS1 with a p.Glu549 variant in Puerto Rican patients [7]. Founder effects are well-described in genetically isolated populations such as the Caribbean, where population bottlenecks and limited admixture may amplify specific alleles [7].

While BBS1 mutations have been associated with relatively milder systemic and ocular disease compared to BBS7 and BBS10 [6], our findings indicate that homozygous BBS1 variants can still result in significant visual morbidity. Patients with BBS1 mutations have shown worse BCVA and more pronounced macular thinning compared to those with compound heterozygous states, supporting the concept that allelic variation and zygosity play critical roles in determining disease severity [8]. By separately evaluating homozygous and compound heterozygous BBS1 cases, this study highlights how allelic configuration influences retinal structure and visual function. This approach may help refine prognostic counseling and allow for more tailored monitoring in clinical practice.

The severity and early onset of visual decline in this cohort reflect the underlying ciliopathy pathophysiology of BBS. Photoreceptor cells are highly dependent on intraflagellar transport mediated by the BBSome protein complex. Disruption of this transport impairs the delivery of essential proteins between the inner and outer segments of photoreceptors, leading to progressive dysfunction and cell death [12].

Our findings reinforce the value of macular OCT and automated visual field testing as complementary structural and functional disease measures in patients with BBS. Given the variability in ERG availability, structural OCT parameters and automated visual fields were prioritized as the most consistent and reproducible measures. These modalities allowed for standardized comparisons between genotypes and remain highly informative in BBS, where macular thinning and peripheral field loss closely correlate with disease severity. Use of a standardized macular cube protocol on the Cirrus platform strengthened the comparability of OCT-derived metrics within the cohort. Future multicenter studies should aim to harmonize imaging protocols to enable cross-population genotype-phenotype analyses.

Electrophysiological testing (ERG) was not included in this analysis because it was inconsistently available across the study period and missing for most patients, preventing meaningful genotype-specific comparisons. However, this represents an important functional measure in ciliopathies, and future studies incorporating full-field ERG may provide additional insight into retinal function beyond structural assessment. Similarly, as with many retrospective inherited retinal disease cohorts, ancillary testing such as fundus autofluorescence imaging or kinetic perimetry was not uniformly available. This heterogeneity underscores the need for prospective standardized protocols in future BBS studies.

The correlation between macular thickness/volume and mean deviation (MD) mirrors prior evidence in inherited retinal diseases showing that retinal thinning parallels functional decline [13,14]. Given their reproducibility and non-invasive nature, these modalities should be prioritized for longitudinal monitoring and could provide surrogate endpoints in future interventional trials.

Clinically, this study underscores the need for genotype-phenotype surveillance in BBS. Patients with homozygous BBS1 mutations may require closer ophthalmic follow-up and targeted counseling regarding visual prognosis. Incorporating OCT and visual field metrics into routine care can aid in quantifying disease progression and may facilitate earlier interventions.

Limitations of this study include its retrospective design, modest sample size, absence of longitudinal follow-up, and incomplete availability of ancillary testing such as ERG and fundus autofluorescence. Despite these constraints, this represents one of the largest genetically characterized BBS cohorts in Puerto Rico. Future work should focus on prospective, multicenter studies across the Caribbean to validate these findings and expand the understanding of the genetic architecture of BBS in underrepresented populations. Longitudinal tracking of visual function and retinal structure will also be critical for developing prognostic genotype-phenotype models and for preparing the field for future gene- or cell-based therapies.

## Conclusions

This study highlights the predominance of BBS1 mutations and their strong association with severe visual impairment in a Puerto Rican cohort with BBS. The marked structural and functional retinal degeneration observed in homozygous BBS1 patients underscores the importance of genotype-informed surveillance and tailored management strategies. Incorporating OCT and visual field testing as standardized biomarkers may enhance clinical monitoring and readiness for future therapies. These findings further emphasize the need for early genetic diagnosis and population-specific research to advance precision care in underrepresented groups.
